# Association between Structural Connectivity and Generalized Cognitive Spectrum in Alzheimer’s Disease

**DOI:** 10.3390/brainsci10110879

**Published:** 2020-11-20

**Authors:** Angela Lombardi, Nicola Amoroso, Domenico Diacono, Alfonso Monaco, Giancarlo Logroscino, Roberto De Blasi, Roberto Bellotti, Sabina Tangaro

**Affiliations:** 1 Istituto Nazionale di Fisica Nucleare, Sezione di Bari, 70125 Bari, Italy; angela.lombardi@ba.infn.it (A.L.); nicola.amoroso@uniba.it (N.A.); domenico.diacono@ba.infn.it (D.D.); roberto.bellotti@ba.infn.it (R.B.); 2 Dipartimento di Farmacia–Scienze del Farmaco, Università degli Studi di Bari, 70125 Bari, Italy; 3 Center for Neurodegenerative Diseases and the Aging Brain, Università degli Studi di Bari at Pia Fondazione “Card. G. Panico”, 73039 Tricase, Italy; giancarlo.logroscino@uniba.it; 4 Department of Basic Medicine Neuroscience and Sense Organs, Università degli Studi di Bari, 70124 Bari, Italy; 5 Pia Fondazione “Card. G. Panico”, 73039 Tricase, Italy; robertodeblasi@hotmail.com; 6 Dipartimento Interateneo di Fisica, Università degli Studi di Bari, 70126 Bari, Italy; 7 Dipartimento di Scienze del Suolo, della Pianta e degli Alimenti, Università degli Studi di Bari, 70126 Bari, Italy

**Keywords:** alzheimer’s disease, biomarker identification, machine learning, brain connectivity, diffusion tensor imaging

## Abstract

Modeling disease progression through the cognitive scores has become an attractive challenge in the field of computational neuroscience due to its importance for early diagnosis of Alzheimer’s disease (AD). Several scores such as Alzheimer’s Disease Assessment Scale cognitive total score, Mini Mental State Exam score and Rey Auditory Verbal Learning Test provide a quantitative assessment of the cognitive conditions of the patients and are commonly used as objective criteria for clinical diagnosis of dementia and mild cognitive impairment (MCI). On the other hand, connectivity patterns extracted from diffusion tensor imaging (DTI) have been successfully used to classify AD and MCI subjects with machine learning algorithms proving their potential application in the clinical setting. In this work, we carried out a pilot study to investigate the strength of association between DTI structural connectivity of a mixed ADNI cohort and cognitive spectrum in AD. We developed a machine learning framework to find a generalized cognitive score that summarizes the different functional domains reflected by each cognitive clinical index and to identify the connectivity biomarkers more significantly associated with the score. The results indicate that the efficiency and the centrality of some regions can effectively track cognitive impairment in AD showing a significant correlation with the generalized cognitive score (*R* = 0.7).

## 1. Introduction

Alzheimer’s disease (AD) is a widespread neurodegenerative disease that causes irreversible and progressive memory loss, resulting in the decline of intellectual and social skills [1]. The early stages of AD are characterized by mild memory problems, hence great efforts have been done in investigating effective markers for early diagnosis of the disease in order to improve care and treatment pathways or find innovative personalized drug therapies [2,3]. Moreover, the amnestic onset in AD is by large the most prevalent, but it is important to note that atypical onset (with frontal and posterior cortical variants) have an incidence of about 20% [4].

Several tests provide information about the neuropsychological conditions of patients and measure the severity of the most important symptoms of AD. The most commonly used cognitive indices include: Alzheimer’s Disease Assessment Scale cognitive total score (ADAS), Mini Mental State Exam score (MMSE) and Rey Auditory Verbal Learning Test (RAVLT) which measures cognitive impairment, attention, language and visuospatial functions and memory deficits. Such scores provide a quantitative assessment of the cognitive conditions of the patients, and they are used as objective criteria for clinical diagnosis of dementia [5,6]. As a matter of fact, modeling disease progression through the cognitive scores has become an attractive challenge in the field of computational neuroscience due to its importance for early diagnosis of AD [7,8].

The AD progression can also be accurately observed by using magnetic resonance imaging (MRI). Several MRI features have been associated with cognitive scores in AD such as average regional cortical thickness, white matter (WM) volume [9], cortical surface area, tissue volume and gray matter density (GM) [10,11,12]. Early works focused on simple regression models to predict selected cognitive outcomes. More recently, several studies have proposed multivariate learning methods in order to improve the predictive performance and identify the most relevant imaging biomarkers [13].

An ever-increasing number of works is dedicated to the study of brain connectivity in Alzheimer’s disease [14]. Indeed, the recent literature highlights that the AD decline is associated to disrupted connectivity among brain regions caused by degeneration of white matter (WM) [15]. In particular, diffusion weighted imaging (DWI) has become the most popular technique to investigate the physical connection among WM fibers, i.e., the structural connectivity [16,17]. DWI and tractography algorithms are combined to define diffusion tensor imaging (DTI) structural networks that could be analyzed through complex network models [18,19,20]. This approach involves modeling the brain as a network of anatomical regions linked by WM fiber tracts. Hence, connectivity patterns could be investigated by using several topological network metrics describing the roles of the regions, the structure of paths connecting them and their importance for the network integrity [21]. Very accurate classification of AD and MCI subjects has been achieved by combining complex network modeling and machine learning (ML) algorithms proving the potential applications of structural DTI networks in the clinical setting [22,23,24].

We carried out a pilot study to investigate the strength of association between DTI structural connectivity and cognitive spectrum in Alzheimer’s disease. Our hypothesis is based on the assumption that, if some structural connectivity patterns are efficiently used to classify groups of pathological and MCI subjects, then connectivity configurations could exist to shape the cognitive decline spectrum. Our goal is predicting the cognitive decline on a continuum range of values instead of using distinct diagnostic labels in order to better characterizes the cognitive changes at individual level. Hence, we firstly summarized the cognitive domains by using a single generalized data-driven score. Then, we used a machine learning framework with the twofold aim to: (i) test the strength of association between the cognitive score and the structural connectivity of subjects with a broad spectrum of decline; and (ii) identify the biomarkers of structural connectivity more significantly associated with the generalized score.

## 2. Materials

### 2.1. Subjects

A dataset from the Alzheimer’s Disease Neuroimaging Initiative (ADNI) database (http://adni.loni.usc.edu/) was used in this work. In 2003, ADNI was initiated as a multi-site longitudinal study involving multiple biological markers and clinical and neuropsychological tests to determine the progression of early AD. The goal of recognizing responsive and precise markers of AD progression is to help researchers to develop new therapies and monitor their validity, as well as to reduce costs and length of clinical trials. The images analyzed for this study belong to 191 subjects, both male and female. In accordance with the diagnosis, the subjects were grouped into 48 normal controls (NC) (age 73.4±5.7), 39 AD patients (age 75.4±8.8) and 104 MCI converter subjects (age 72.8±7.4), i.e., MCI that converted to AD from 3 months to 5 years after the date of scan. NC subjects show no signs of depression, MCI or dementia. Participants with AD are those who meet the NINCDS/ADRDA criteria for probable AD. MCI subjects have reported a subjective memory concern, but without any significant impairment in other cognitive domains: they substantially preserved everyday activities with no sign of dementia.

Each subject underwent a cognitive assessment including mini-mental state examination test (MMSE) (scores below 24 indicate impairment) and Alzheimer’s disease assessment scale (ADAS) (scores less than or equal to 10 may be considered in the normal range) and other tests described in Section 2.2. Demographic information and clinical scores for the participants are listed in Table 1. The diffusion-weighted scans were acquired using a 3T GE Medical Systems scanner. For each subject, we considered both T1-weighted 3D anatomical spoiled gradient echo (SPGR) sequences (256×256 matrix; voxel size =1.2×1.0×1.0 mm${}^{3}$; TI =400 ms; TR =6.98 ms; TE =2.85 ms; flip angle $=11^{\circ }$) and diffusion weighted images (256×256 matrix with a field of view of 35 cm; voxel size =2.7×2.7×2.7 mm${}^{3}$; scan time =9 min; repetition time/echo time =9 s/60 ms; flip angle $=90^{\circ }$). More specifically, 46 separate DWI images were acquired for each scan: 5 with negligible diffusion effects ($b_{0}$ images) and 41 diffusion-weighted images (b = 1000 s/mm${}^{2}$). More details can be found at: http://adni.loni.usc.edu/wp-content/uploads/2010/05/ADNI2_GE_3T_22.0_T2.pdf.

### 2.2. Cognitive Assessment

We considered 24 clinical measures available for each participant in the ADNI Neuropsychological Battery table. This table includes multiple cognitive and functional assessments about memory deficits and behavioral symptoms commonly used as screening tools for detecting dementia and AD. Since some cognitive batteries correlate significantly with each others, we performed a correlation analysis in order to retain only indices with a mutual correlation coefficient R<0.75. Finally, we included the following S=10 clinical measures in the outcome matrix *Y* of our analysis: CDR-SOB, ADAS-Cog-13, MMSE, MoCA, FAQ, RAVLT-immediate, RAVLT-learning, RAVLT-percforgetting, ECog-PT-total and ECog-SP-total. They are detailed as follows:

- Clinical Dementia Rating Scale Sum of Boxes (CDR-SOB) is the index most used in clinical practice for evaluating disease severity including the mild and early symptomatic stages of dementia. The CDR is obtained through semistructured interviews of patients and informants, and cognitive functioning is rated in six functional domains: memory, home and hobbies, personal care, judgment and problem solving, community affairs and orientation. Each domain is rated on a 5-point scale: 0, no impairment; 0.5, questionable impairment; 1, mild impairment; 2, moderate impairment; and 3, severe impairment. The final CDR-SOB score is obtained by summing each of the domain box scores, with scores ranging from 0 to 18 [25].
- The cognitive subscale (ADAS-Cog-11) comprises 11 tasks that include both subject-completed tests and observer-based assessments. The tasks assess cognitive functioning of memory, praxis and language. Specific tasks comprise Naming Objects, Word Recall, Fingers, Commands, Orientation, Word Recognition, Constructional Praxis, Ideational Praxis and Language [26]. The extended version, i.e., the ADAS-Cog-13, includes all ADAS-Cog-11 items as well as a test of delayed word recall and a number cancellation or maze task [27].
- The mini-mental state examination (MMSE) assesses various cognitive domains, including memory, attention and language. Scores for MMSE range from 0 to 30; lower scores indicate greater cognitive dysfunction [28].
- The Montreal cognitive assessment (MoCA) consists of 12 individual tasks (grouped into cognitive domains, including visuospatial and executive functioning, attention, language, abstraction, naming, delayed memory recall and orientation), most of which are binary, and are scored and summed with a 6-item orientation screening and an educational correction to generate a total score representing the global cognitive functioning [29].
- The Functional Activities Questionnaire (FAQ) rates the instrumental activities of daily living (IADLs), such as preparing meals and managing personal finances [30]. The sum scores range in the 0–30 interval and the cut-point of 9 (dependent in 3 or more activities) is recommended to indicate possible cognitive impairment.
- The Rey auditory verbal learning test (RAVLT) consists of five presentations of a 15-word list (List A), each followed by attempted recall. This is followed by a second 15-word interference list (List B), followed by recall of List A. It scores different aspects of episodic memory such as the learning rate (learning and immediate RAVLT) but also delayed recall (forgetting and percent forgetting RAVLT) [31].
- The Everyday Cognition (ECog) scale is an informant-rated questionnaire that includes one global factor and six domain-specific factors. The psychometric properties in the ECog scale focus on everyday function and cognition mild problems reported from both both participant (ECog-PT) and study partner (ECog-SP) [32].

## 3. Methods

### 3.1. Image Processing

The following steps were performed to reconstruct the brain connectivity of each subject from the raw DWI scans. First, we acquired the raw DICOM images from the ADNI database. We converted the DICOM images into the NIFTI format by using the MRIcron suite (https://www.nitrc.org/projects/mricron). Then, we organized the NIFTI images into the BIDS standard format. We executed all the processing steps by using the MRtrix3 software package (http://mrtrix.org [33,34]), including scripts interacting with the package FSL FMRIB Software Library (FSL) https://fsl.fmrib.ox.ac.uk/fsl/fslwiki/ [35] for some steps. In detail, the DWI images were processed by performing some standard steps, as described in our previous works [24,36]. First, we applied a denoising step to enhance the signal-to-noise ratio (SNR) of the MR signals. The FSL’s eddy correct tool was applied to correct the head motion and eddy distortion in each subject by performing alignment of the DWI scans to the average $b_{0}$ image. Skull-stripping was done with the brain extraction tool (BET) [37]. A correction field was firstly estimated from the $b_{0}$ image, and then we applied the bias-field correction to all volumes. The software tool fsl_anat was applied to process the T1-weighted anatomical scans and re-orient them to the standard image MNI152. The inter-modal registration between the T1-weighted anatomical image and the DWI for each subject was the last step.

We applied the connectome pipeline to generate the structural connectome by firstly generating a tissue-segmented image [38] and then by applying unsupervised estimation of gray matter, white matter and cerebro-spinal fluid response functions. The fiber orientation distributions (FOD) for spherical deconvolution [39] was finally estimated. To generate the probabilistic tractography [40] using both dynamic seeding [41] and anatomically-constrained tractography (ACT) [42], the Spherical-deconvolution Informed Filtering of Tractograms (SIFT2) methodology was applied [41]. Finally, we mapped the resulting streamlines through an anatomical parcellation scheme using the AAL2 atlas [43] with 120 regions obtaining the connectivity matrix from the streamlines file and the atlas.

### 3.2. Network Metrics

A 120×120 weighted symmetric connectivity matrix *W* was obtained for each subject as output of the image processing steps; the entry $w_{ij}$ of *W* represents the number of fiber tracts connecting region *i* to region *j*.

Several topological metrics exist to assess the importance of the regions with respect to the rest of the network. In this pilot study, we chose to explore the most intuitive metrics to quantitatively describe the centrality and influence of the network of each region. In detail, the following graph metrics were extracted from each matrix *W* and for each node of the network i=1,…,N, with N=120:

- The node strength is a direct measure of centrality that characterize the relative importance of a node in a network by considering the weights of all the links of a node:
  (1) $$s_{i}=\sum_{j=1}^{N}w_{ij}$$
- Eigenvector centrality assesses the influence of a node in a self-referential manner by computing the centrality for a node based on the centrality of its neighbors [44]:
  (2) $$eig_{i}=\frac{1}{\Lambda }\sum_{j=1}^{N}w_{ij}{eig}_{j},$$
  where Λ is the largest eigenvalue associated with the eigenvector of the matrix *W*.
- The local efficiency of a node $E(G_{i})$ characterizes how well information is exchanged by its neighbors when it is removed [45]:
  (3) $$E\left(G_{i}\right)=\frac{1}{n_{i}\left(n_{i}-1\right)}\sum_{j\in G_{i}}\frac{1}{d(i,j)},$$
  where $G_{i}$ is the local subgraph including the immediate neighbors of the node *i*, $n_{i}$ denotes the total nodes in the subgraph $G_{i}$ and d(i,j) denotes the length of the shortest path between the node *i* and another neighbor node *j* obtained by minimizing the sum of the weights of the links connecting the two nodes.

We used the MATLAB implementation of Brain Connectivity Toolbox (BCT) [21] to compute the graph metrics. We finally constructed two matrices of features:

1. The $M\times P_{1}$, ($M=191,P_{1}=7140$) matrix $X_{1}$ has as features the elements of the upper triangular matrix *W* of each subject. Thus, this matrix includes the connectivity-related weights.
2. The $M\times P_{2}$, ($M=191,P_{2}=360$) matrix $X_{2}$ has as features the three network metrics (e.g., strength, eigenvector centrality and efficiency) for each ROI. Accordingly, each feature is labeled as: “graph metric—roi”. This matrix describes the central role of each brain region.

### 3.3. Machine Learning Framework

Here, we developed a machine learning framework to:

1. identify a generalized index that effectively summarizes the cognitive spectrum of the population under investigation;
2. find significant associations between the identified index and the features derived from the structural connectivity of the subjects; and
3. identify the most important features in order to understand the strongest biological associations between the structural connectivity and cognitive spectrum.

**Figure 1:**
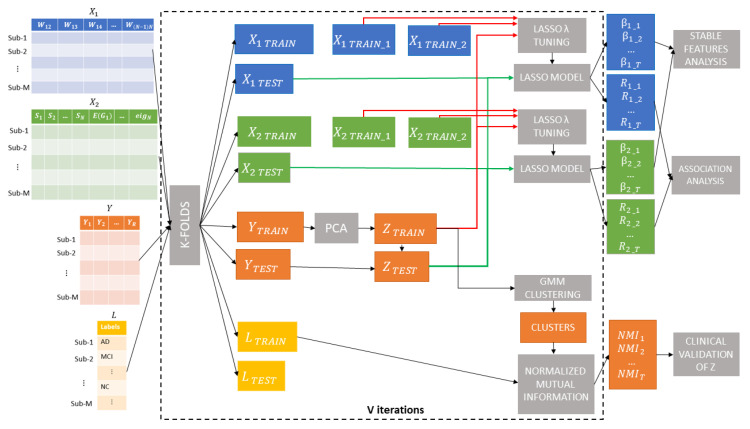
Machine learning framework: V=10 re-sampling of a k-fold (k=10) cross-validation were executed, producing T=100 subsets of $X_{1}$, $X_{2}$ and *Y* datasets. In each iteration, nine-folds of the score matrix (i.e., $Y_{TRAIN}$) were input to principal component analysis (PCA) to find a single generalized cognitive score $Z_{TRAIN}$ from the ten clinical indices. A Gaussian Mixture Models (GMM) clustering analysis was executed to compare the data-driven partitions with the diagnosis labels $L_{TRAIN}$ for each subject provided in the ADNI Neuropsychologica Battery table. Within each cross-validation round, a Lasso regression model was trained for each of the two datasets $X_{1_{TRAIN}}$ and $X_{2_{TRAIN}}$. The two trained models were tested on the left out fold $X_{1_{TEST}}$ and $X_{2_{TEST}}$ to predict $Z_{TEST}$ and evaluate the most effective connectivity dataset. The weights $\beta_{ij}$ of the best Lasso models were employed to identify the particular subset of features that yields the best performance.

The main steps are shown in Figure 1. Briefly, V=10 re-sampling of a k-fold (k=10) cross-validation was executed, producing T=100 subsets of $X_{1}$, $X_{2}$ and *Y* datasets. In each iteration, nine folds of the score matrix (i.e., $Y_{TRAIN}$) were input to the first module to find a single generalized cognitive score $Z_{TRAIN}$ from the ten clinical indices by means of principal component analysis (PCA). The new score was validated by using a clustering-based analysis in conjunction with the diagnosis labels $L_{TRAIN}$ for each subject provided in the ADNI Neuropsychological Battery table. Within each cross-validation round, we trained a Lasso regression model for each dataset $X_{1_{TRAIN}}$ and $X_{2_{TRAIN}}$. We finally tested the two trained model on the left out fold $X_{1_{TEST}}$ and $X_{2_{TEST}}$ to predict $Z_{TEST}$ and evaluate the most effective connectivity dataset. We also used the weights $\beta_{ij}$ of the best Lasso models to identify the particular subset of features that yields the best performance by means of a stability analysis [46]. Each step of the framework is detailed in the next sections.

#### 3.3.1. Identification and Validation of the Generalized Cognitive Score

We applied PCA [47] to find a comprehensive cognitive score from a set of partially correlated clinical variables while accounting for the maximum percentage of their total variance. PCA has been broadly adopted in clinical applications to reduce the dimensionality of large datasets in an interpretable way, while preserving the statistical information of the data [48,49]. Thus, we computed the first component of the matrix of the clinical scores within each training fold (i.e., $Y_{TRAIN}$) to obtain the vector of the generalized scores $Z_{TRAIN}$ for the training samples as:

(4) $$Z_{TRAIN}=Y_{TRAIN}*c_{1}$$

where $c_{1}$ is the corresponding vector of coefficients of the first component.

Then, the generalized scores $Z_{TEST}$ for the test data were computed by using the coefficients obtained from the PCA of the training dataset:

(5) $$Z_{TEST}=Y_{TEST}*c_{1}$$

The contribution of each variable to the explained variance of the first principal component across the rounds was quantified to describe the final composition of the generalized score.

Moreover, we performed a clustering analysis to provide a clinical validation of the computed score, as follows:

- Gaussian mixture models (GMM) were adopted to model $Z_{TRAIN}$ as a mixture of unimodal distributions in which each mode corresponds to a different subpopulation [50]. This clustering algorithm involves the Expectation Maximization (EM) technique for automatically estimating the parameters of the individual distribution components [51].
- The normalized mutual information (NMI) [52] between the set of identified subpopulations and the set of diagnostic labels (e.g., NC, AD and MCI) for the training samples was computed to assess the percentage of overlap between the two partitions.

**Figure 2:**
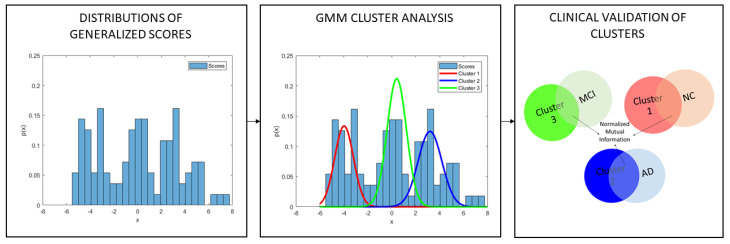
Illustrative example of clinical validation of the generalized scores resulting from PCA in a single round: a cluster analysis with gaussian mixture models (GMM) was conducted to decompose the distributions of scores into a mixture of unimodal distributions in which each mode corresponds to a different cluster. The normalized mutual information (NMI) between the set of identified clusters and the set of diagnostic labels (e.g., NC, AD and MCI) was used to assess the percentage of overlap between the identified clusters and the clinical groups.

This procedure shown in Figure 2 returns a metric of reliability of the new generalized score in the diagnostic domain since the NMI value indicates the level of agreement between the clusters identified in an unsupervised manner by using only the score and the corresponding diagnostic labels of the subjects belonging to each cluster. We also performed a permutation test to assess the statistical significance of the overlap between the identified clusters and the clinical groups. In detail, a null distribution was generated by randomizing group labels 10,000 times and by calculating the NMI value between the GMM clusters and the permuted labels at each permutation. Finally, a *p* value was assigned as the number of times that the permuted overlap was greater than the actual NMI values, divided by the number of permutations. We used the MATLAB GMM implementation provided by the Statistics and Machine Learning Toolbox (https://it.mathworks.com/products/statistics.html).

#### 3.3.2. Association between Connectivity and Generalized Cognitive Score

Lasso (Least Absolute Shrinkage and Selection Operator) [53] was employed to find significant associations between the connectivity features and the proposed generalized cognitive score. Indeed, Lasso is a regularization method that was introduced to solve both overfitting and multicollinearity problems in ordinary least square regression. This approach involves a penalty term that controls the complexity of the model by introducing sparsity. This term penalizes the coefficients of the least significant variables shrinking some of them to zero so only the most important features are retained. The outcome is a subset of the predictors that contribute mostly to the regression model, so the algorithm is also used as embedded feature selection method. The goal of this method is to minimize the residual sum of squares (RSS) to find the coefficients of the predictors:

(6) $$RSS=\frac{1}{2}\left|\right|Z_{TRAIN}-\beta X_{TRAIN}{\left|\right|}_{2}^{2}-{\lambda ||\beta ||}_{1}$$

The λ parameter should be tuned for the optimization of the accuracy. We divided the training set $X_{TRAIN}$ within each round into a training and validation set to find the best value of λ. Hence, a single model was trained with the dataset $X_{TRAIN}$ within each round and it was tested on the left out fold $X_{TEST}$ to predict $Z_{TEST}$. We used the Statistics and Machine Learning MATLAB Toolbox for Lasso implementation.

Within each round, we evaluated the performance of the model through the correlation coefficient between the actual values of the generalized cognitive score $z_{i}$ of $Z_{TEST}$ and the model’s predicted values $\hat{z_{i}}$:

(7) $$R=\frac{\sum_{i=1}^{M}\left(z_{i}-\bar{z}\right)\left(\hat{z_{i}}-\bar{\hat{z}}\right)}{\sqrt{\sum_{i=1}^{M}{\left(z_{i}-\bar{z}\right)}^{2}}\sqrt{\sum_{i=1}^{M}{\left(\hat{z_{i}}-\bar{\hat{z}}\right)}^{2}}},$$

where $\bar{z}$ and $\bar{\hat{z}}$ denote their sample means.

#### 3.3.3. Identification of Significant Features

The output produced by Lasso within each round of cross-validation is a sparse vector of weights β. We analyzed the matrix $B=[\beta_{1},\ldots ,\beta_{T}]$ of size T×P, being *T* the number of iterations and *P* the number of features. Since the matrix *B* is sparse, we considered only entries with non-zero weight as features relevant to the target variable within each round of cross validation. Consequently, we analyzed both the stability and the relevance of the features by applying a frequency-based criterion and a threshold-based selection of their weights across the rounds. In particular, to identify the most repeatable features among all the rounds, we selected only the features whose frequency was greater than 99% percentile of the frequency distribution. In fact, a feature selection algorithm might be sensitive with respect to changes in the training set, yielding subsets of features not representative of the overall population under investigation [54]. The assessment of the stability of the selected features over the rounds was thus carried out to select the list of more stable features with respect to small changes in the training sets taken from the whole sample distribution [55,56].

## 4. Results

### 4.1. Identification and Clinical Validation of the Generalized Cognitive Score

**Figure 3:**
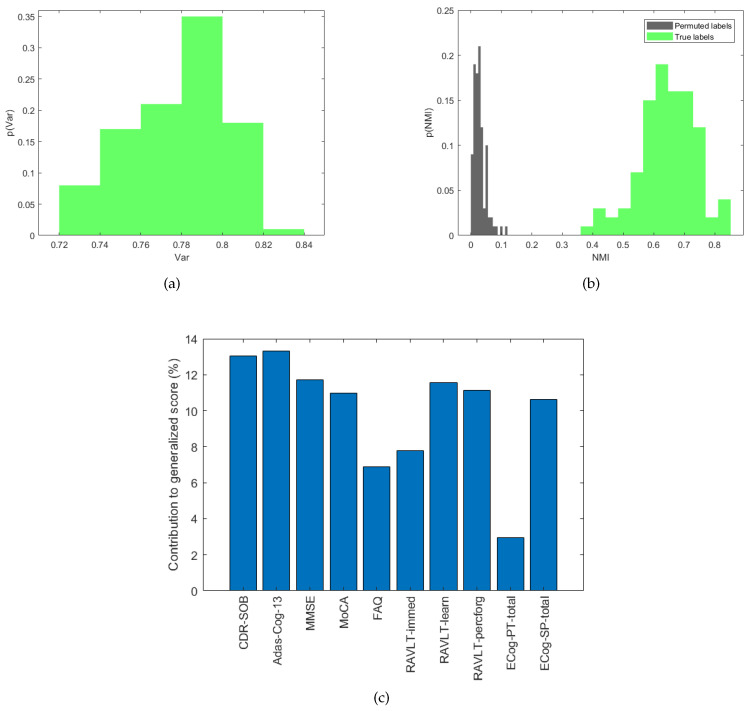
(**a**) Empirical probability distribution of the variance explained by the first component of the ten indices. (**b**) Empirical probability distributions of NMI values for the actual labels and permuted labels. (**c**) Contribution of each clinical index to the explained variance of the generalized score averaged across the validation rounds.

We performed the PCA of the ten cognitive scores in each round of cross-validation as described in Section 3.3.1. As shown in Figure 3a, the average percentage of explained variance of the first component over all the rounds is Var=0.78±0.02. This result suggests that the first latent variable may explain the relationship between the ten observed variables, and it can therefore reasonably represent a generalized cognitive score.

Then, we used the GMM algorithm to identify the optimal partition of the cognitive score into clusters. We measured the overlap between the GMM clusters and the clinical groups by computing the NMI score within each cross-validation round. The distributions of the actual NMI values and the null distribution are shown in Figure 3b. A significant overlap between the GMM clusters and the clinical groups was obtained with an average NMI value =0.65±0.09 (p=0). Figure 3c represents the average contribution of each clinical index to the generalized score. It is possible to note that almost all the clinical indices contribute in the same amount to the generalized score, except for FAQ, RAVLT-immed and ECog-PT-Total, which show a contribution below 8%.

### 4.2. Best Model Selection

**Figure 4:**
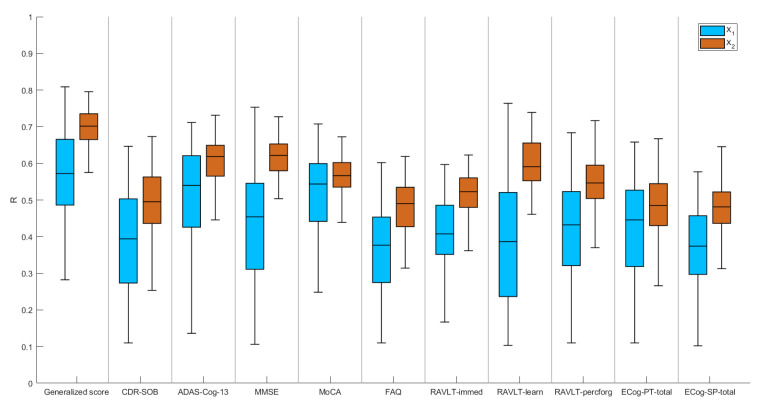
Correlation between the actual values and the values predicted by Lasso algorithm of the generalized cognitive score and each of the ten clinical indices by using both connectivity weights (matrix $X_{1}$) and local graph metrics (matrix $X_{2}$) as features.

Figure 4 shows the boxplots of the correlation coefficient between the actual values of the generalized cognitive score and the predicted values resulting from the Lasso algorithm by using both the connectivity features, i.e., the connectivity weights (matrix $X_{1}$) and the local connectivity metrics (matrix $X_{2}$). To compare the performance achieved with the proposed generalized cognitive score, we also performed the association analysis with each of the ten clinical indices. Table 2 summarizes the mean and standard deviation quantities of the distributions of correlation coefficients.

As clearly shown in Figure 4 and Table 2, higher performance is achieved by using the generalized cognitive score in comparison to each of the clinical indices. We also performed a Wilcoxon rank-sum test to compare the distributions of R values resulting from the generalized score and the other clinical indices finding that the generalized score performed significantly better than the others for both matrices $X_{1}$ and $X_{2}$ (p<0.0001 for each comparison). Moreover, the model with the graph metrics as features shows significantly greater performance (average R=0.7±0.05, Wilcoxon rank-sum test p<0.0001) than the model that employs the entire structural connectivity matrix (average R=0.57±0.11), highlighting that a local description of the connectivity of each individual could adequately predict the generalized cognitive score. We found λ=0.62±0.1 for $X_{1}$ and λ=0.25±0.1 for $X_{2}$ across the rounds.

### 4.3. Identification of Significant Features

The relative frequency of the selected features and their average weights ($\beta_{i}$) were evaluated across the validation rounds to rank the importance of each feature for the most performing matrix $X_{2}$.

**Figure 5:**
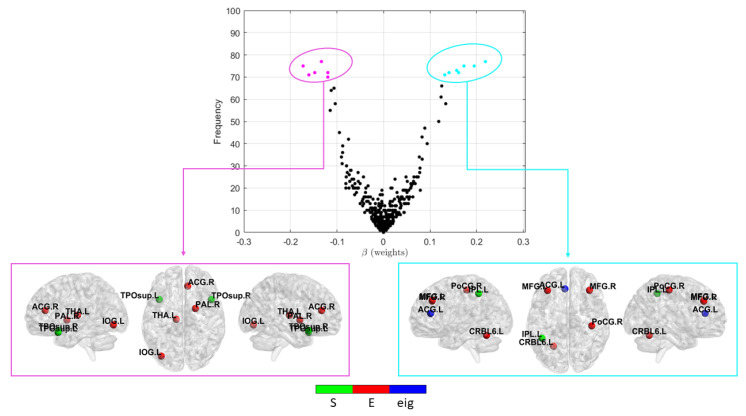
(**Top**) Weights vs. stability of the $X_{2}$ features across all the validation rounds; (**bottom left**) the most stable features with the highest negative associations with the generalized cognitive score; and (**bottom right**) the most stable features with the highest positive associations with the generalized cognitive score.

As can be noticed in Figure 5, a strong correlation exists between stability, i.e., greater frequency of occurrence in different validation rounds, and average weights of features resulting from the Lasso models. In particular, the most stable features (i.e., those with an occurrence greater than 70%) for both negative and positive association with the generalized cognitive score are shown in the bottom part of Figure 5. In Table 3, the same regions with their MNI coordinates are also specified. It is worth noting that, for both positive and negative associations, the nodal efficiency of the regions prevails over the other two graph metrics.

## 5. Discussion

In this study, we combined all the cognitive clinical indices of a mixed ADNI cohort of AD, MCI and NC subjects in a data driven manner to extract a generalized score reflecting multiple cognitive domains. The final composition of the generalized index showed in Figure 3c indicates that almost all clinical indices contribute in the same way to the generalized index, highlighting the importance of multiple cognitive domains covered by the tests and clinical questionnaires. We clinically validated the new generalized score by using an unsupervised approach. Our results show that the new score explains a percentage of variance of all the indices greater than 70% and that the partitions identified with the clustering algorithm significantly overlap with the clinical labels in the database as shown in Figure 3. Then, we applied a machine learning regression algorithm to: (i) investigate the strength of association between the generalized score and the structural connectivity of the subjects; and (ii) identify the connectivity features most related to the generalized score. We also investigated the association between the connectivity patterns and each of the clinical index included in the ADNI database. Our analysis revealed a stronger association between structural connectivity and generalized score than between the other clinical indices, showing the highest correlation index between the actual and predicted scores (R=0.7). It is important to note that this score was introduced to summarize the different cognitive functional domains, maximizing the variance of the different clinical indices in order to test the association between the topological organization of structural connectivity and a single quantitative index through a regression algorithm. However, as shown in Table 2, both Adas-Cog and MMSE indices exhibit significant associations with the structural connectivity indices, reporting the same performance (R=0.6).

These two indices are widely recognized as the most reliable for the cognitive assessment in the clinical setting. The associations of these two indices with some MRI biomarkers have been explored in other works. In [11], relevance vector regression (RVR) algorithm based on different feature extraction approaches was used to predict MMSE scores by using tissue density maps extracted from the MRI images of an ADNI cohort of 122 subjects. They found a correlation between the estimated and the measured MMSE scores around 0.73 obtained by using regional features. Zhang et al. [12] adopted a joint-prediction strategy to estimate two regression variables (MMSE and ADAS-Cog) and one classification variable (i.e., clinical label) of 186 ADNI subjects, from the baseline MRI, PET and CSF data achieving R=0.697±0.022 for MMSE prediction and R=0.739±0.012 for ADAS-Cog prediction. Similar results were achieved in [57,58,59,60] by using a joint regression and classification approach and multimodal imaging. Such approaches are focused on clinical classification of the subjects, hence combine clinical label and clinical scores to build a more robust classification model by taking into account the information of the relation between the high-level clinical label and clinical scores as well as the relation among samples in the feature selection step. More recently, Huang et al. [61] outperformed other state-of-art regression models by using normalized volumes of 90 brain regions together with a nonlinear sparse learning model version of random forest (RF) to predict CDR-SOB, MMSE and ADAS-cog scores. These works underline some important aspects of disease staging in AD: (i) more complex nonlinear models could better characterize real relationship between the applied features and the clinical scores; and (ii) multimodal imaging could considerably improve the accuracy of machine learning models by providing complementary information to better recognize neurodegenerative patterns. We carried out a preliminary exploratory analysis of the association between the structural connectivity derived from the single DTI modality and a new score representative of the maximum information content of the ten clinical indices. For this reason, we adopted a less complex model in favor of a direct interpretability of the features more strongly related to the regression task. Indeed, both $l_{1}$ and $l_{2}$ norm regularization methods were extensively employed to extract features that have impact on the clinical scores, based on the assumption that a given imaging marker can affect multiple cognitive scores and only a subset of the features are relevant for the regression task [62]. These models are often used to perform feature selection as sparsity is imposed by regularization methods to extract the most important predictors [63,64]. It is important to underline that a critical interpretation of the results must take into account the different dimensionality of the datasets (i.e., $P_{1}>>P_{2}$). A high number of superfluous predictors may lead to models that overfit the training data and fail to generalize on the test data, even when regularization algorithms are adopted [65]. Further investigations could clarify the worst performance achieved with the dataset $X_{1}$. As an example, more objective comparisons between the two datasets could be reached by including more subjects in the analysis.

In addition, we performed a stability analysis on the features selected across all validation rounds in order to derive the most significant set of features for the generalized score prediction task, regardless of the subpopulation extracted in a specific validation fold. As shown in Figure 5, this stability analysis revealed only few stable structural connectivity markers significantly associated to the generalized score. In particular, bilateral superior temporal pole, right anterior cingulate cortex and left inferior occipital were found negatively associated with the proposed score. Volumetric reduction, atrophy increase and WM integrity changes of the cingulate, occipital gyrus and temporal pole have been found in AD progression [66,67,68,69]. In several studies, DTI brain networks were used to analyze the main differences between some topological metrics in the AD, NC and MCI groups. In particular, decreased global efficiency and reduced nodal efficiency in cingulate cortex and several prefrontal regions were found in AD and amnestic MCI [16,70]. These findings are consistent with our results that show a strength decrease of both left and right superior temporal pole and reduced efficiency in the other regions. Moreover, as shown in Table 3, the efficiency metric prevails over the other two graph metric, evidencing that this metric could better capture the cognitive decline in AD. The local efficiency measures the ability of information exchange of the subnetwork consisting of itself and its direct neighbors, thus it could detect the nodes that play a key role in the information integration [71]. Interestingly, we also found features positively associated with the generalized cognitive score, i.e., their values increase, as the cognitive impairment increases. Such findings could be related to structural reorganization of brain connectivity and compensatory processes such as resilience mechanisms to cognitive decline [72]. It is noteworthy that our work explicitly addresses an association analysis between structural connectivity and cognitive spectrum. However, numerous studies have exploited functional magnetic resonance imaging to correlate functional connectivity to a patient’s cognitive status [73]. A future study using both modalities could better highlight functional and structural connectivity patterns associated with the cognitive spectrum in AD.

## 6. Conclusions

In this study, we found that a generalized cognitive score obtained in a data-driven manner by combining the available clinical cognitive scores is more significantly associated with structural connectivity. In particular, we showed that some local topological descriptors of structural connectivity can effectively track cognitive impairment in Alzheimer’s disease. These promising results suggest that structural DTI networks contain clinically relevant information about cognitive function and can be developed into biomarkers to describe cognitive decline associated with AD. In future work, we will test more complex nonlinear machine learning models on a larger data sample to further investigate the relationship between the cognitive status and the topological organization of the structural connectivity networks in a more heterogeneous aging population.

## Figures and Tables

**Table 1:** Demographic and clinical scores (mean ± standard deviation) of the study participants.

	NC (48)	AD (39)	MCI (104)
Age	73.4±5.7	75.4±8.8	72.8±7.4
Gender	24 M/24 F	26 M/13 F	64 M/40 F
CDR-SOB	0.04±0.13	4.8±1.3	1.41±0.7
Adas-Cog 13	9±4.7	30.9±8.7	15.9±6.7
MMSE	29±1.1	23±1.8	27±1.6
MoCA	25.6±2.1	17.5±4.3	22.9±2.7
FAQ	0.23±0.92	15.1±6.9	2.7±4
RAVLT-immed	44.3±10.4	21.3±6.7	34.1±9.6
RAVLT-learn	5.12±2.3	2±1.9	4.3±2.1
RAVLT-percforg	36.2±28.2	89±19.4	56.8±32
ECog-PT-total	1.2±0.2	1.9±0.6	1.8±0.5
ECog-SP-total	1.2±0.3	2.8±0.5	1.7±0.6

(CDR-SOB, Clinical Dementia Rating Scale Sum of Boxes; ADAS-Cog-13, Cognitive subscale 13; MMSE, Mini-Mental State Examination; MoCA, Montreal Cognitive Assessment; FAQ, Functional Activities Questionnaire; RAVLT-immed, Rey Auditory Verbal Learning Test-immediate; RAVLT-learn, Rey Auditory Verbal Learning Test-learning; RAVLT-percforg, Rey Auditory Verbal Learning Test-percent forgetting; ECog-PT-total, Everyday Cognition participant; ECog-SP-total, Everyday Cognition Study Partner). The subjects were grouped into 48 normal controls (NC), 39 Alzheimer’s disease (AD) patients and 104 Mild Cognitive Impairment (MCI) subjects. The database consisted of 114 males (M) and 77 females (F).

**Table 2:** Mean and standard deviation quantities of the distribution of correlation coefficients between the actual values of each clinical index and the values predicted by Lasso regression algorithm.

Clinical Index	$X_{1}$	$X_{2}$
Generalized score	0.57±0.11	0.70±0.05
CDR-SOB	0.39±0.14	0.49±0.09
Adas-Cog 13	0.51±0.13	0.60±0.07
MMSE	0.42±0.15	0.60±0.07
MoCA	0.50±0.13	0.56±0.06
FAQ	0.36±0.11	0.47±0.07
RAVLT-immed	0.40±0.10	0.51±0.07
RAVLT-learn	0.39±0.18	0.59±0.07
RAVLT-percforg	0.42±0.14	0.54±0.07
ECog-PT-total	0.41±0.14	0.48±0.08
ECog-SP-total	0.37±0.11	0.47±0.08

**Table 3:** Most stable features and their association with the generalized cognitive score.

ROIs	Abbreviation (MNI Coordinates)	Graph Metric	Association
Right anterior cingulate gyrus	ACG.R (8.46, 37.01, 15.84)	E	negative
Left inferior occipital gyrus	IOG.L (−36.36, −78.29, −7.84)	E	negative
Right pallidum	PAL.R (21.2, 0.18, 0.23)	E	negative
Left thalamus	THAL.L (−10.85, −17.56, 7.98)	E	negative
Left superior temporal gyrus	TPOsup.L (−39.88, 15.14, −20.18)	S	negative
Right superior temporal gyrus	TPOsup.R (48.25, 14.75, −16.86)	S	negative
Left middle frontal gyrus	MFG.L (−33.43, 32.73, 35.46)	E	positive
Right middle frontal gyrus	MFG.R (37.59, 33.06, 34.04)	E and eig	positive
Left anterior cingulate gyrus	ACG.L (−4.04, 35.4, 13.95)	eig	positive
Right postcentral gyrus	PoCG.R (41.43, −25.49, 52.55)	E	positive
Left inferior parietal gyrus	IPL.L (−42.8, −45.82, 46.74)	S	positive
Left cerebellum 6	CRBL6.L (−23.24, −59.10, −22.13)	E	positive

## References

[1] Association A. (2019). 2019 Alzheimer’s disease facts and figures. Alzheimers Dement..

[2] Morris J.C., Storandt M., Miller J.P., McKeel D.W., Price J.L., Rubin E.H., Berg L. (2001). Mild cognitive impairment represents early-stage Alzheimer disease. Arch. Neurol..

[3] Latta C.H., Brothers H.M., Wilcock D.M. (2015). Neuroinflammation in Alzheimer’s disease; a source of heterogeneity and target for personalized therapy. Neuroscience.

[4] Dubois B., Feldman H.H., Jacova C., Hampel H., Molinuevo J.L., Blennow K., DeKosky S.T., Gauthier S., Selkoe D., Bateman R. (2014). Advancing research diagnostic criteria for Alzheimer’s disease: The IWG-2 criteria. Lancet Neurol..

[5] Teng E., Becker B.W., Woo E., Knopman D.S., Cummings J.L., Lu P.H. (2010). Utility of the Functional Activities Questionnaire for distinguishing mild cognitive impairment from very mild Alzheimer’s disease. Alzheimer Dis. Assoc. Disord..

[6] Kueper J.K., Speechley M., Montero-Odasso M. (2018). The Alzheimer’s disease assessment scale–cognitive subscale (ADAS-Cog): Modifications and responsiveness in pre-dementia populations. a narrative review. J. Alzheimers Dis..

[7] Hsu J.L., Hsu W.C., Chang C.C., Lin K.J., Hsiao T., Fan Y.C., Bai C.H. (2017). Everyday cognition scales are related to cognitive function in the early stage of probable Alzheimer’s disease and FDG-PET findings. Sci. Rep..

[8] Moradi E., Hallikainen I., Hänninen T., Tohka J., Alzheimer’s Disease Neuroimaging Initiative (2017). Rey’s Auditory Verbal Learning Test scores can be predicted from whole brain MRI in Alzheimer’s disease. NeuroImage Clin..

[9] Zhou J., Liu J., Narayan V.A., Ye J., Alzheimer’s Disease Neuroimaging Initiative (2013). Modeling disease progression via multi-task learning. NeuroImage.

[10] Stonnington C.M., Chu C., Klöppel S., Jack C.R., Ashburner J., Frackowiak R.S., Alzheimer’s Disease Neuroimaging Initiative (2010). Predicting clinical scores from magnetic resonance scans in Alzheimer’s disease. NeuroImage.

[11] Wang Y., Fan Y., Bhatt P., Davatzikos C. (2010). High-dimensional pattern regression using machine learning: From medical images to continuous clinical variables. NeuroImage.

[12] Zhang D., Shen D., Alzheimer’s Disease Neuroimaging Initiative (2012). Multi-modal multi-task learning for joint prediction of multiple regression and classification variables in Alzheimer’s disease. NeuroImage.

[13] Cao P., Liu X., Liu H., Yang J., Zhao D., Huang M., Zaiane O. (2018). Generalized fused group lasso regularized multi-task feature learning for predicting cognitive outcomes in Alzheimers disease. Comput. Methods Programs Biomed..

[14] Amoroso N., La Rocca M., Bruno S., Maggipinto T., Monaco A., Bellotti R., Tangaro S. (2018). Multiplex networks for early diagnosis of Alzheimer’s disease. Front. Aging Neurosci..

[15] Rose S.E., Chen F., Chalk J.B., Zelaya F.O., Strugnell W.E., Benson M., Semple J., Doddrell D.M. (2000). Loss of connectivity in Alzheimer’s disease: An evaluation of white matter tract integrity with colour coded MR diffusion tensor imaging. J. Neurol. Neurosurg. Psychiatry.

[16] Lo C.Y., Wang P.N., Chou K.H., Wang J., He Y., Lin C.P. (2010). Diffusion tensor tractography reveals abnormal topological organization in structural cortical networks in Alzheimer’s disease. J. Neurosci..

[17] Amoroso N., Monaco A., Tangaro S., Neuroimaging Initiative (2017). Topological measurements of DWI tractography for Alzheimer’s disease detection. Comput. Math. Methods Med..

[18] Bullmore E., Sporns O. (2009). Complex brain networks: Graph theoretical analysis of structural and functional systems. Nat. Rev. Neurosci..

[19] Amico E., Goñi J. (2018). Mapping hybrid functional-structural connectivity traits in the human connectome. Netw. Neurosci..

[20] Tipnis U., Amico E., Ventresca M., Goni J. (2018). Modeling communication processes in the human connectome through cooperative learning. IEEE Trans. Netw. Sci. Eng..

[21] Rubinov M., Sporns O. (2010). Complex network measures of brain connectivity: Uses and interpretations. NeuroImage.

[22] Ebadi A., Dalboni da Rocha J.L., Nagaraju D.B., Tovar-Moll F., Bramati I., Coutinho G., Sitaram R., Rashidi P. (2017). Ensemble classification of Alzheimer’s disease and mild cognitive impairment based on complex graph measures from diffusion tensor images. Front. Neurosci..

[23] Lella E., Amoroso N., Lombardi A., Maggipinto T., Tangaro S., Bellotti R., Alzheimer’s Disease Neuroimaging Initiative (2019). Communicability disruption in Alzheimer’s disease connectivity networks. J. Complex Netw..

[24] Lella E., Lombardi A., Amoroso N., Diacono D., Maggipinto T., Monaco A., Bellotti R., Tangaro S. (2020). Machine learning and dwi brain communicability networks for alzheimer’s disease detection. Appl. Sci..

[25] O’Bryant S.E., Waring S.C., Cullum C.M., Hall J., Lacritz L., Massman P.J., Lupo P.J., Reisch J.S., Doody R. (2008). Staging dementia using Clinical Dementia Rating Scale Sum of Boxes scores: A Texas Alzheimer’s research consortium study. Arch. Neurol..

[26] Rosen W.G., Mohs R.C., Davis K.L. (1984). A new rating scale for Alzheimer’s disease. Am. J. Psychiatry.

[27] Mohs R.C., Knopman D., Petersen R.C., Ferris S.H., Ernesto C., Grundman M., Sano M., Bieliauskas L., Geldmacher D., Clark C. (1997). Development of cognitive instruments for use in clinical trials of antidementia drugs: Additions to the Alzheimer’s Disease Assessment Scale that broaden its scope. Alzheimer Dis. Assoc. Disord..

[28] Folstein M.F., Folstein S.E., McHugh P.R. (1975). “Mini-mental state”: A practical method for grading the cognitive state of patients for the clinician. J. Psychiatr. Res..

[29] Nasreddine Z.S., Phillips N.A., Bédirian V., Charbonneau S., Whitehead V., Collin I., Cummings J.L., Chertkow H. (2005). The Montreal Cognitive Assessment, MoCA: A brief screening tool for mild cognitive impairment. J. Am. Geriatr. Soc..

[30] Pfeffer R.I., Kurosaki T.T., Harrah C., Chance J.M., Filos S. (1982). Measurement of functional activities in older adults in the community. J. Gerontol..

[31] Schmidt M. (1996). Rey Auditory Verbal Learning Test: A Handbook.

[32] Farias S.T., Mungas D., Reed B.R., Cahn-Weiner D., Jagust W., Baynes K., DeCarli C. (2008). The measurement of everyday cognition (ECog): Scale development and psychometric properties. Neuropsychology.

[33] Tournier J.D., Calamante F., Connelly A. (2012). MRtrix: Diffusion tractography in crossing fiber regions. Int. J. Imaging Syst. Technol..

[34] Tournier J.D., Smith R., Raffelt D., Tabbara R., Dhollander T., Pietsch M., Christiaens D., Jeurissen B., Yeh C.H., Connelly A. (2019). MRtrix3: A fast, flexible and open software framework for medical image processing and visualisation. NeuroImage.

[35] Jenkinson M., Beckmann C.F., Behrens T.E., Woolrich M.W., Smith S.M. (2012). Fsl. Neuroimage.

[36] Lella E., Amoroso N., Diacono D., Lombardi A., Maggipinto T., Monaco A., Bellotti R., Tangaro S. (2019). Communicability characterization of structural DWI subcortical networks in Alzheimer’s disease. Entropy.

[37] Smith S.M. (2002). Fast robust automated brain extraction. Hum. Brain Mapp..

[38] Zhang Y., Brady M., Smith S. (2001). Segmentation of brain MR images through a hidden Markov random field model and the expectation-maximization algorithm. IEEE Trans. Med. Imaging.

[39] Jeurissen B., Tournier J.D., Dhollander T., Connelly A., Sijbers J. (2014). Multi-tissue constrained spherical deconvolution for improved analysis of multi-shell diffusion MRI data. NeuroImage.

[40] Tournier J.D., Calamante F., Connelly A. Improved probabilistic streamlines tractography by 2nd order integration over fibre orientation distributions. Proceedings of the International Society for Magnetic Resonance in Medicine (Ismrm).

[41] Smith R.E., Tournier J.D., Calamante F., Connelly A. (2015). SIFT2: Enabling dense quantitative assessment of brain white matter connectivity using streamlines tractography. NeuroImage.

[42] Smith R.E., Tournier J.D., Calamante F., Connelly A. (2012). Anatomically-constrained tractography: Improved diffusion MRI streamlines tractography through effective use of anatomical information. NeuroImage.

[43] Rolls E.T., Joliot M., Tzourio-Mazoyer N. (2015). Implementation of a new parcellation of the orbitofrontal cortex in the automated anatomical labeling atlas. NeuroImage.

[44] Bonacich P. (1987). Power and centrality: A family of measures. Am. J. Sociol..

[45] Latora V., Marchiori M. (2001). Efficient behavior of small-world networks. Phys. Rev. Lett..

[46] Lombardi A., Guaragnella C., Amoroso N., Monaco A., Fazio L., Taurisano P., Pergola G., Blasi G., Bertolino A., Bellotti R. (2019). Modelling cognitive loads in schizophrenia by means of new functional dynamic indexes. NeuroImage.

[47] Joliffe I., Morgan B. (1992). Principal component analysis and exploratory factor analysis. Stat. Methods Med. Res..

[48] Zhang Z., Castelló A. (2017). Principal components analysis in clinical studies. Ann. Transl. Med..

[49] Hansen L.K., Larsen J., Nielsen F.Å., Strother S.C., Rostrup E., Savoy R., Lange N., Sidtis J., Svarer C., Paulson O.B. (1999). Generalizable patterns in neuroimaging: How many principal components?. NeuroImage.

[50] Reynolds D.A. (2009). Gaussian Mixture Models. Encyclopedia of Biometrics.

[51] Yang M.S., Lai C.Y., Lin C.Y. (2012). A robust EM clustering algorithm for Gaussian mixture models. Pattern Recognit..

[52] Kuncheva L.I., Hadjitodorov S.T. Using diversity in cluster ensembles. Proceedings of the 2004 IEEE International Conference on Systems, Man and Cybernetics (IEEE Cat. No. 04CH37583).

[53] Tibshirani R. (1996). Regression shrinkage and selection via the lasso. J. R. Stat. Soc. Ser. B (Methodol.).

[54] Kalousis A., Prados J., Hilario M. (2007). Stability of feature selection algorithms: A study on high-dimensional spaces. Knowl. Inf. Syst..

[55] Li X., Wang X., Xiao G. (2019). A comparative study of rank aggregation methods for partial and top ranked lists in genomic applications. Briefings Bioinform..

[56] Lombardi A., Amoroso N., Diacono D., Monaco A., Tangaro S., Bellotti R. (2020). Extensive Evaluation of Morphological Statistical Harmonization for Brain Age Prediction. Brain Sci..

[57] Zhu X., Suk H.I., Shen D. (2014). A novel matrix-similarity based loss function for joint regression and classification in AD diagnosis. NeuroImage.

[58] Yu G., Liu Y., Shen D. (2016). Graph-guided joint prediction of class label and clinical scores for the Alzheimer’s disease. Brain Struct. Funct..

[59] Zhu X., Suk H.I., Wang L., Lee S.W., Shen D., Alzheimer’s Disease Neuroimaging Initiative (2017). A novel relational regularization feature selection method for joint regression and classification in AD diagnosis. Med. Image Anal..

[60] Liu M., Zhang J., Adeli E., Shen D. (2018). Joint classification and regression via deep multi-task multi-channel learning for Alzheimer’s disease diagnosis. IEEE Trans. Biomed. Eng..

[61] Huang L., Jin Y., Gao Y., Thung K.H., Shen D., Alzheimer’s Disease Neuroimaging Initiative (2016). Longitudinal clinical score prediction in Alzheimer’s disease with soft-split sparse regression based random forest. Neurobiol. Aging.

[62] Liu J., Ji S., Ye J. (2012). Multi-task feature learning via efficient l2, 1-norm minimization. arXiv.

[63] Wang H., Nie F., Huang H., Risacher S., Ding C., Saykin A.J., Shen L. Sparse multi-task regression and feature selection to identify brain imaging predictors for memory performance. Proceedings of the IEEE 2011 International Conference on Computer Vision.

[64] Gui J., Sun Z., Ji S., Tao D., Tan T. (2016). Feature selection based on structured sparsity: A comprehensive study. IEEE Trans. Neural Netw. Learn. Syst..

[65] Hawkins D.M. (2004). The problem of overfitting. J. Chem. Inf. Comput. Sci..

[66] Jones B.F., Barnes J., Uylings H.B., Fox N.C., Frost C., Witter M.P., Scheltens P. (2006). Differential regional atrophy of the cingulate gyrus in Alzheimer disease: A volumetric MRI study. Cereb. Cortex.

[67] Wang L., Goldstein F.C., Veledar E., Levey A.I., Lah J.J., Meltzer C.C., Holder C.A., Mao H. (2009). Alterations in cortical thickness and white matter integrity in mild cognitive impairment measured by whole-brain cortical thickness mapping and diffusion tensor imaging. Am. J. Neuroradiol..

[68] Bosch B., Arenaza-Urquijo E.M., Rami L., Sala-Llonch R., Junqué C., Solé-Padullés C., Peña-Gómez C., Bargalló N., Molinuevo J.L., Bartrés-Faz D. (2012). Multiple DTI index analysis in normal aging, amnestic MCI and AD. Relationship with neuropsychological performance. Neurobiol. Aging.

[69] Fu Z., Iraji A., Caprihan A., Adair J.C., Sui J., Rosenberg G.A., Calhoun V.D. (2020). In search of multimodal brain alterations in Alzheimer’s and Binswanger’s disease. NeuroImage Clin..

[70] Daianu M., Jahanshad N., Nir T.M., Jack C.R., Weiner M.W., Bernstein M.A., Thompson P.M., Alzheimer’s Disease Neuroimaging Initiative (2015). Rich club analysis in the Alzheimer’s disease connectome reveals a relatively undisturbed structural core network. Hum. Brain Mapp..

[71] Van den Heuvel M.P., Sporns O. (2013). Network hubs in the human brain. Trends Cogn. Sci..

[72] Allen G.I., Amoroso N., Anghel C., Balagurusamy V., Bare C.J., Beaton D., Bellotti R., Bennett D.A., Boehme K.L., Boutros P.C. (2016). Crowdsourced estimation of cognitive decline and resilience in Alzheimer’s disease. Alzheimers Dement..

[73] Binnewijzend M.A., Adriaanse S.M., Van der Flier W.M., Teunissen C.E., de Munck J.C., Stam C.J., Scheltens P., van Berckel B.N., Barkhof F., Wink A.M. (2014). Brain network alterations in Alzheimer’s disease measured by eigenvector centrality in fMRI are related to cognition and CSF biomarkers. Hum. Brain Mapp..

